# Molecular Dynamics Simulations Reveal the Interaction Fingerprint of Remdesivir Triphosphate Pivotal in Allosteric Regulation of SARS-CoV-2 RdRp

**DOI:** 10.3389/fmolb.2021.639614

**Published:** 2021-08-20

**Authors:** Mitul Srivastava, Lovika Mittal, Anita Kumari, Shailendra Asthana

**Affiliations:** Translational Health Science and Technology Institute (THSTI), Faridabad, India

**Keywords:** SARS-CoV-2 RNA-dependent RNA polymerase (RdRp), RDV triphosphate (RTP), molecular dynamics simulation, PCA, FEL, NTP entrance site, allosteric inhibition, remdesivir

## Abstract

The COVID-19 pandemic has now strengthened its hold on human health and coronavirus’ lethal existence does not seem to be going away soon. In this regard, the optimization of reported information for understanding the mechanistic insights that facilitate the discovery towards new therapeutics is an unmet need. Remdesivir (RDV) is established to inhibit RNA-dependent RNA polymerase (RdRp) in distinct viral families including Ebola and SARS-CoV-2. Therefore, its derivatives have the potential to become a broad-spectrum antiviral agent effective against many other RNA viruses. In this study, we performed comparative analysis of RDV, RMP (RDV monophosphate), and RTP (RDV triphosphate) to undermine the inhibition mechanism caused by RTP as it is a metabolically active form of RDV. The MD results indicated that RTP rearranges itself from its initial RMP-pose at the catalytic site towards NTP entry site, however, RMP stays at the catalytic site. The thermodynamic profiling and free-energy analysis revealed that a stable pose of RTP at NTP entrance site seems critical to modulate the inhibition as its binding strength improved more than its initial RMP-pose obtained from docking at the catalytic site. We found that RTP not only occupies the residues K545, R553, and R555, essential to escorting NTP towards the catalytic site, but also interacts with other residues D618, P620, K621, R624, K798, and R836 that contribute significantly to its stability. From the interaction fingerprinting it is revealed that the RTP interact with basic and conserved residues that are detrimental for the RdRp activity, therefore it possibly perturbed the catalytic site and blocked the NTP entrance site considerably. Overall, we are highlighting the RTP binding pose and key residues that render the SARS-CoV-2 RdRp inactive, paving crucial insights towards the discovery of potent inhibitors.

## Introduction

SARS-CoV-2 genome consists of a (+) ssRNA virus and its RNA-dependent RNA polymerase (RdRp) which help in viral replication and synthesis of its genome (Eastman et al., 2020; Gao et al., 2020). RdRp has a high sequence and structural conservation that makes it an attractive drug target for various RNA viruses diseases (Mittal et al., 2019, 2020; Gao et al., 2020; Maddipati et al., 2020). Currently, RDV is one of the most promising drugs for COVID-19 being evaluated in phase III clinical trials in China and the United States (Eastman et al., 2020). It was originally developed to treat Ebola virus and possess a broad-spectrum activity (Babadaei et al., 2020). RDV is a prodrug that consists of nucleoside analogue scaffold and it is metabolised into an alanine metabolite (GS-704277) inside the cells, then gets converted into the monophosphate derivative (RMP) and eventually into the active nucleoside triphosphate derivative (RTP) (Eastman et al., 2020). The nucleotide analog drugs have been a cornerstone of antiviral and anticancer therapy that target viral polymerases that cause chain termination. Nucleotide analogs are not highly permeable to cells, and once within the cell they need di- and then tri-phosphorylation to generate the nucleoside triphosphate (NTP) which could be used as a mimic of natural NTPs (competing the natural substrates) of genome replication by viral RNA-dependent polymerases (Chen et al., 2014; Eastman et al., 2020). Such inhibitors will initially reach the hepatic cells as non-phosphorylated or monophosphate prodrugs, after which cellular kinases turn them into active triphosphate (Fung et al., 2014). The viral RdRp will then mis-incorporate these incoming inhibitors as NTPs into their viral RNA strand, eventually leading to the chain termination process (Eastman et al., 2020). Generally, nucleoside TP analogs lack a 3′OH group that causes complete chain termination; however, RTP has this 3′OH group but also consists of a 1′CN group that delays the mechanism of chain termination (Eastman et al., 2020). The 1′CN group of RTP is reported to sterically clash with residue S861 of RdRp that affects the chain elongation step (Eastman et al., 2020).

Recently, the crystal structure of SARS-CoV-2 RdRp in complex with RMP, two cofactors NSP7 and NSP8, and primer-template strands were released in Protein Data Bank (PDB-ID: 7BV2), showing intermolecular interactions between RMP, primer-template, and RdRp (Kato et al., 2020). It was determined using cryo-electron microscopy which inhabits all 932 amino acids (Kato et al., 2020; Yin et al., 2020). It contains an N-terminal β-hairpin (residues 31–50) and an extended nidovirus RdRp-associated nucleotidyl-transferase domain (NiRAN, residues 115–250), composed of seven folded helices with three β-strands. Additionally, there is an interface domain (residues 251–365), which consists of three helices and five β-strands, that is further connected to the RdRp domain (residues 366–920) (Yin et al., 2020) (Supplementary Figure S1). The RdRp is surrounded by three subdomains: a fingers subdomain (residues L366 to A581 and K621 to G679), a palm subdomain (residues T582 to P620 and T680 to Q815), and a thumb subdomain (residues H816 to E920) (Supplementary Figure S1). It also contains seven evolutionary conserved motifs, such as in other HCV, DENV, BVDV, and poliovirus (PV) RdRps, which perform specific and essential functions during polymerization (Gong and Peersen, 2010; Appleby et al., 2015; Mittal et al., 2020). These are termed as motifs A–G, out of which the finger domain consists of two important motifs, G (residue 499–511) and F (residue F544-560), whereas the palm domain contains a catalytic core of the RdRp and four conserved motifs A to D, and partially motif E (motif A: residues 611–626; motif B: residue 678–710, motif C: residues 753–767, motif D: residues 771–796, motif E: residues 810–820) (Zhang et al., 2020). The motif A contains the conserved residue D618 which is classic divalent-cation–binding and motif C consists of conserved catalytic residues ^759^SDD^761^ (Yin et al., 2020). Motifs F (residues K545 and R555) and G (residues K500 and S501) are involved in interaction with RNA template strand and escort it to the active site (catalytic site) (Yin et al., 2020). The residues involved in RNA binding as well as the residues comprising the catalytic active site are highly conserved, highlighting the preserved mechanism of RdRp genome replication in these various RNA viruses, and indicating that wide spectrum antiviral inhibitors could be created.

The arrival of SARS-CoV-2 RdRp crystal structures of bound RMP (PDB ID: 7BV2) and APO (PDB ID: 7BV1) has provided an opportunity to elucidate their complex behavior and to understand the comprehensive inhibition mechanism. The recent studies are based on understanding the dynamicity of the modeled RdRp and its interaction with either RDV (Koulgi et al., 2020) or RTP (Zhang and Zhou, 2020). In the modeled RdRp, RDV and RTP are stated to interact strongly at the catalytic site and NTP entry sites, respectively (Zhang and Zhou, 2020). However, neither RDV nor RMP are the active forms but RTP is known to completely inhibit the polymerization activity of RdRp (Yin et al., 2020). Hence, our aim is to understand the interaction pattern of RTP when it binds to the target in comparison with RDV and RMP as it is the active form and is able to render the RdRp inactive. Therefore, our objective is to elucidate the mechanistic details of RTP. With this objective, we are aiming to provide a comparative insight in terms of interaction fingerprinting to identify the key residues and their pivotal role in the mechanism of inhibition. Their dynamic and energetic contributions were mapped through MD simulations in terms of dynamical and thermodynamical components. Further, we have implemented the PCA and FEL analysis to highlight the major conformational changes and consequently the porcupine plot distinguished the activity of these molecules on the basis of differential atomic motions. The comprehensive analysis offers a rational for RTP being the active state of RDV as it fully overshadows the NTP entry channel and gains favorable contact with key residues which are crucial for NTP entry and other residues, namely K545, R553, R555, D618, P620, K621, R624, K798, and R836. These residues contribute significantly in holding RTP at the NTP entrance site and therefore perturbing the replication process. Apart from these residues the conservation in allosteric sites and SiteMap analysis was also carried out in BVDV, DENV, and HCV RdRps that could further be exploited. Based on these observations and understanding that highlight the mechanism of inhibition as well as the structural level insights, further structural-functional studies are recommended. The study provides scope to target these residues for the discovery of potential non-nucleoside inhibitors (NNIs).

## Materials and Methods

### System Preparation

The protein structure of the SARS-CoV-2 RdRp was retrieved from the RCSB protein data bank (PDB ID: 7BV2, 7BV1). We also included crystal structures of HCV, BVDV, and DENV RdRp (PDB-ID:4NLD (for T1 and P2 site), 3FRZ (for T2 site), 4EAW (P1 site), 5I3Q (Thumb and Palm junction), and 3FRZ, 5IQ6, and 1S48 (for Template entrance site) (Mittal et al., 2019, 2020; Maddipati et al., 2020) to identify the probable allosteric sites in RdRp protein employed on the basis of a literature survey. The protein structure was prepared using the Protein Preparation Wizard module of Maestro (Sastry et al., 2013; Anang et al., 2018) (Schrödinger release 2020–1: Maestro, Schrödinger, LLC, New York, NY, 2020.) (Sarkar et al., 2021). OPLS3 (Jorgensen et al., 1996) force field model was used for preparation (Jorgensen et al., 1996). It has been found that the crystal structure has several breaks from K50-E84, D100-R118 (regions of NiRAN domain), and M906-E919 (region of RdRp domain). To maintain the structural integrity during dynamics, all loop breaks were interpolated using the PLOP algorithm (Srivastava et al., 2018). The loop conformations were optimized and again the structure was minimized. In the crystal structure, RDV monophosphate (RMP) is bound to RdRp rather than the RDV triphosphate (RTP), where the later is known to be the active form. Hence, RDV along with its metabolites RMP and RTP were prepared using LigPrep module for further study (LigPrep, version 4.2; Schrödinger, LLC, New York, NY, 2020–1). The optimization was done using the OPLS3 force field (Jorgensen et al., 1996).

### Molecular Docking

To understand the broader spectrum of the catalytic site as well as the NTP entry site, we included RDV as a parent molecule in our study. Since no bound crystal with RDV is yet reported, we performed targeted docking (Asthana et al., 2013; Asthana et al., 2014) of RDV as well as RTP on the grid coordinates of RMP (bound in crystal 7BV2). Afterwards, to identify different binding modes, focused docking was performed by taking the best conformer achieved from targeted docking. We have included targeted docking as initial coordinates of RMP were available and afterwards, to enrich the conformations and to explore any other binding modes, the focused docking was implemented. The docking experiments were performed using AUTODOCK4.2 (Trott and Olson, 2010; Tyagi et al., 2020; Singh et al., 2021).

### Molecular Dynamic Simulations

All-atom MD simulations of each system were performed by using AMBER16 software suite (Case et al., 2005; Tyagi et al., 2021). The systems were initially subjected for 100ns each while the docked system of RTP was further extended to 100ns to obtain its convergence. The systems (COM-RMP, COM-RDV, and COM-RTP) were simulated, including catalytic site Mg^2+^ ions. Model systems by Allner et al. and Carlson et al. were used for ions and triphosphate group, respectively (Meagher et al., 2003; Allnér et al., 2012). The parameters for RTP compatible with force fields were derived from the RED server (Vanquelef et al., 2011). The systems were solvated inside an orthorhombic box of TIP3P waters with a 12 Å padding in each direction. In leap, AMBERff14SB force field (Maier et al., 2015) was assigned to proteins, hydrogens were added, and counter ions were added to neutralize the system. The systems were minimized in three steps for system relaxation and removal of bad contacts. In the first step, 2000 steps minimization was implemented comprising 1,000 steps of steepest descent method followed by 1,000 steps of conjugate gradient. The first and second stages involved the position restraints of 10 and 2 kcal mol^−1^ Å^−2^, respectively, on the whole protein systems to relax the solvent molecules. The third stage of minimization was carried out unrestrained. The SHAKE algorithm is used to constrain all bonds involving hydrogen atoms. The systems were heated for 20 ps from 0–300 K and equilibrated for 500 ps at 300 K and 1 atm pressure. Thereafter, the simulations were performed in the NPT ensemble at a time step of 2.0 ps. The coordinates were saved at every 20 ps and are referred to as ‘frames’ in this study. The details of the simulated systems are mentioned in Supplementary Table S1. For post-MD analysis, CPPTRAJ module was used for quantifying the RMSD (root mean square deviation) of backbone atoms, RMSF (root mean square fluctuation) of Cα atoms, SASA (solvent accessible solvent area), and rGyr (radius of gyration). For the calculation of these values, the starting frame, which represent the crystal pose, was chosen as the reference frame. The hydrogen bonds (HBs) calculation was implemented on the stable tarjectory using HBonds Tcl script with the criteria 3.5 Å for donor-acceptor distance and 180^∘^ for the angle between acceptor, hydrogen, and donor atoms.

### Free Energy Analysis Through MM-GBSA/PBSA Methods

The average binding energy was calculated for equilibrated MD trajectories for COM systems using MM-GBSA/PBSA approaches in Amber16 (Suri et al., 2014; Guo et al., 2012; Chen et al., 2016). For this, the 500 frames were extracted at equal intervals from the last 25 ns trajectory. The binding free energy (ΔG_bind_) on each system is evaluated as follows:

$$\Delta {\text{G}}_{\text{bind}}={\text{G}}_{\text{com}}–\left({\text{G}}_{\text{rec}}+{\text{G}}_{\text{lig}}\right)$$ (1)

where G_com_, G_rec,_ and G_lig_ are the absolute free energies of complex, receptor, and ligand respectively, arranged over the equilibrium trajectory. The free energy, G, for each species can be calculated by using MM-GBSA and MM-PBSA approaches as follows:

$$\text{G}={\text{E}}_{\text{gas}}+{\text{G}}_{\text{sol}}–\text{TS}$$ (2)

$${\text{E}}_{\text{gas}}={\text{E}}_{\mathrm{int}}+{\text{E}}_{\text{ele}}+{\text{E}}_{\text{vdw}}$$ (3)

$${\text{G}}_{\text{polar},\text{PB}\left(\text{GB}\right)}={\text{E}}_{\text{ele}}+{\text{G}}_{\text{sol}–\text{polar},\text{PB}\left(\text{GB}\right)}$$ (4)

$${\text{G}}_{\text{non}–\text{polar},\text{PB}\left(\text{GB}\right)}={\text{E}}_{\text{vdw}}+{\text{G}}_{\text{sol}–\text{np},\text{PB}\left(\text{GB}\right)}$$ (5)

$${\text{G}}_{\text{sol}}={\text{G}}_{\text{PB}\left(\text{GB}\right)}+{\text{G}}_{\text{sol}–\text{np}}$$ (6)

$${\text{G}}_{\text{sol}–\text{np}}=\gamma \text{SAS}$$ (7)

where T and S are the temperature and total solute entropy, respectively. E_gas_ describes the gas phase energy and is the sum of internal energy (E_int_), van der Waals interaction energy (E_vdw_), and electrostatic interaction energy (E_ele_). It is calculated using the parameters described in the AMBERff14SB force field (Maier et al., 2015). G_sol_ is the solvation free energy that can be decomposed into polar and non-polar contributions. G_sol-polar,PB(GB)_ is the polar solvation contribution calculated by solving the Poisson-Boltzmann (PB) and Generalised-Boltzmann (GB) equations (Genheden and Ryde, 2015). Total polar interaction contributions (G_polar,PB(GB)_) were obtained by sum of the E_ele_ and (G_sol-polar,PB(GB)_) (Asthana et al., 2014). G_sol-np_ is the non-polar solvation contribution that was estimated using 0.0072 kcal mol^−1^ Å^−2^ (value of constant γ) and by determining the solvent-accessible surface area (SAS) using a water probe radius of 1.4 Å (Sitkoff et al., 1994). The dielectric constants for solute and solvents were set to 1 and 80 respectively. Total non-polar interaction contributions (G_non-polar,PB(GB)_) were calculated by E_vdw_ and G_sol-np,PB(GB)_ (Asthana et al., 2014). The binding free energy contribution for each residue in protein-ligand complex formation was computed using the GB model using the same number of frames (Suri et al., 2015; Srivastava et al., 2018).

### Electrostatic Potential Calculations

The calculations were done using the APBS program (Baker et al., 2001) in VMD (Humphrey et al., 1996; Mittal et al., 2019) for final complexes achieved by dynamics. The protonated (.pqr) file for both proteins was generated using pdb2pqr module (Dolinsky et al., 2004) and the iso contour value of (+5 kTe^−1^) and (−5 kTe^−1^) was taken for positive and negative potentials, respectively, to generate the iso-surface of the protein.

### Principal Component Analysis and Free Energy Landscape

PCA was implemented to undermine the conformational changes in RdRp protein and especially essential motions were captured in APO and complex systems. The PCA analysis was performed to highlight the essential motions in the proteins (Tripathi et al., 2016; Khan et al., 2017; Mittal et al., 2021). The PCA calculations were performed using the CPPTRAJ program in amber tools and performed over Cα atoms for all the equilibrated systems. In our study, PCA analysis was performed on a stable trajectory after 40 ns. The elements of the positional covariance matrix C is calculated which is defined by the equation below:

$${\text{C}}_{\text{i }}=\text{ <}\left({\text{q}}_{\text{i}}\;-\;\langle {\text{q}}_{\text{i}}\rangle \right)\cdot \left({\text{q}}_{\text{j}}{\;-\text{ <q}}_{\text{j}}\text{>}\right)\text{ >}\left(\text{i, j, }=\;1\text{, }2\text{, }\ldots .3\text{N}\right)$$ (8)

where q_i_ and q_j_ are the Cartesian coordinates of all Cα atoms and N is the number of Cα atoms used in building the matrix C. The < > sign indicates the ensemble average of the atomic positions in the Cartesian space. The resulting principal components (eigenvectors) were ranked by the corresponding total motion captured by them. In the end, there will be 3N eigenvectors generated, where N is the number of Cα atoms used in the building matrix. Porcupine graphs were generated using the “nmode” files obtained after PCA analysis. It basically provides the residue-based mobility plots for each eigenvector. Further, the free energy landscape was performed to explore the conformation change of proteins based on the PCA by using the ‘g_sham’ module (Tripathi et al., 2016; Khan et al., 2017). The free energy, ∆G(X), is calculated by Equation;

$$\Delta \text{G}\left(\text{X}\right)=–{\text{K}}_{\text{B}}\text{T}\;\text{In}\;\text{P}\left(\text{X}\right)$$ (9)

where K_B_ is the Boltzmann constant and T is absolute temperature, X represents the PCs, and P(X) is the probability distribution of the conformation ensemble along the PCs.

### Computational Alanine Scanning

To confirm the *hot-spot* amino acids in the RTP complex, we have performed computational alanine scanning for the residues highlighted in residue-wise energy decomposition results. The calculations were run on 100 frames from the last 100 ns of MD trajectory by using the MM-GBSA approach. In this method, an amino acid of interest is replaced with alanine and relative binding free energy is recalculated. Finally, the difference in the binding free energies of the wild type and mutant, ΔΔG_bind_, was computed as follows:

$$\Delta \Delta {\text{G}}_{\text{bind}}=\Delta {\text{G}}_{\text{bind}}\left[\text{Wild}\;\text{Type}\right]–\Delta {\text{G}}_{bind}\left[\text{Mutant}\right]$$ (10)

Negative values of ΔΔG bind indicate the favorable contributions of residues in wild type while positive values indicate the unfavourable contributions. The mutant models of all the *hot-spot* residues were generated by using the maestro module.

### SiteMap

To map the potential allosteric sites, the SiteMap (Halgren, 2009) module in the Schrodinger suite was used. It identifies putative binding sites by implementing different parameters. The different parameters on the basis of which a potential binding site is considered are: *site score, size, exposure score, enclosure, hydrophobic/hydrophilic character, contact,* and *donor/acceptor character*. As per Halgren’s analysis, the average number of sites for sub-micromolar sites is 132, where lower exposure scores of 0.52 and higher exposure scores of 0.76 on average are considered better for sub-micromolar sites. For the donor/acceptor character and site score, the average for the sub-micromolar sites is 0.76 and 1.01, respectively. Druggability of the site is denoted by Dscore. Dscore values provide a rough estimate of whether the site is druggable. These scores were derived by Halgren (2009) by executing the SiteMap program on a number of proteins that have inhibitors bound with potencies in the sub-micromolar range and performing statistical analyses to produce optimized scores. The OPLS-2003 force field (Jorgensen, 2002) was employed, and a standard grid was used with 15 site points per reported site and cropped at 4.0 Å from the nearest site point.

### Figures

All the images were generated using VMD (Humphrey et al., 1996) and graphs were plotted using XMGRACE (Turner, 2005. XMGRACE, Version 5.1.19. Center for Coastal and Land-Margin Research, Oregon Graduate Institute of Science and Technology, Beaverton, OR; 2005) (Mittal et al., 2020).

## Result and Discussion

### Remdesivir and Remdesivir Triphosphate Binding Mode

**FIGURE 1:**
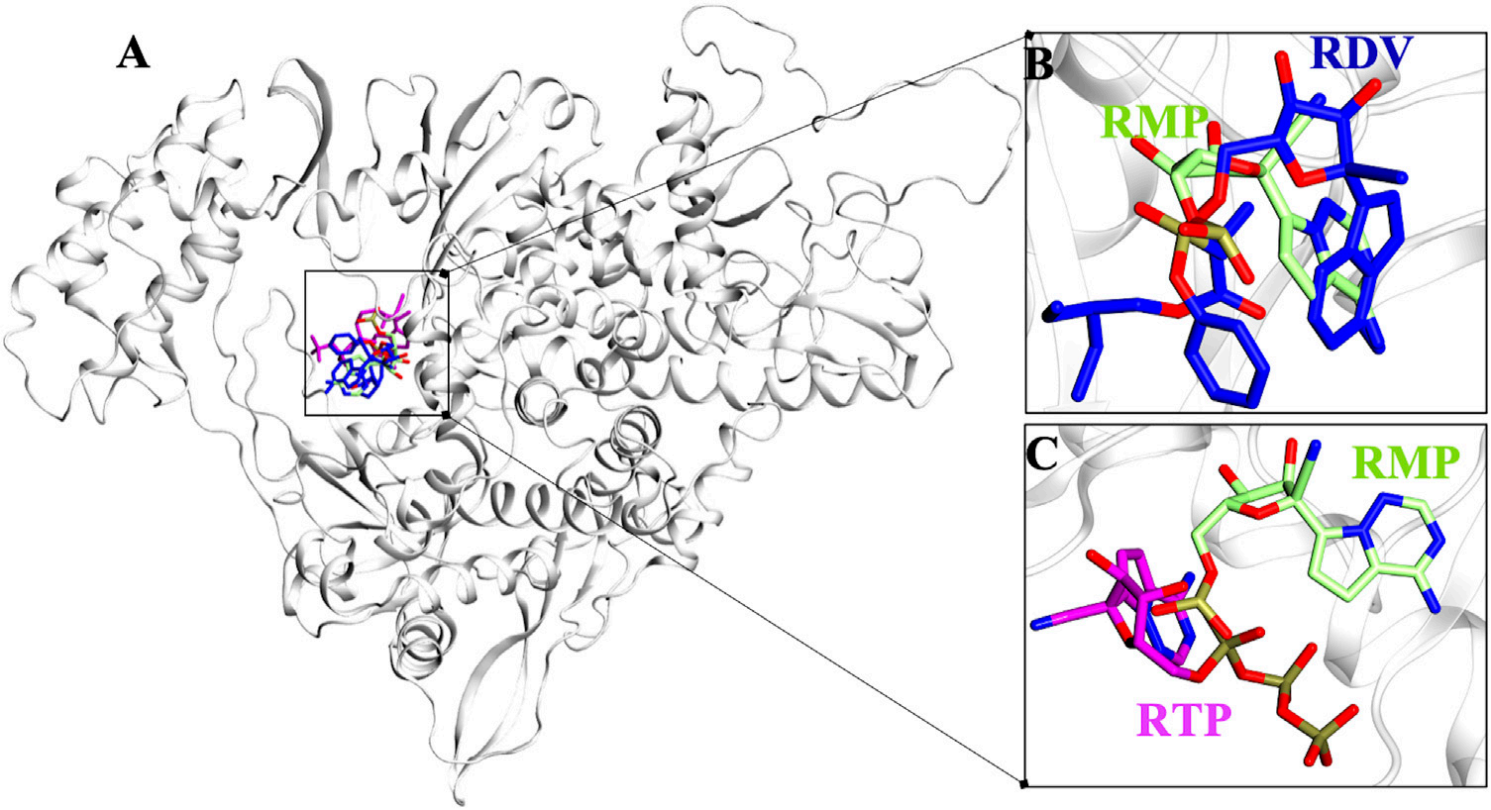
Possible binding pose of RDV and RTP revealed through molecular docking. **(A)** Superimposition of docked pose of RDV and RTP on RMP-crystal pose. The inset shows the docked pose of **(B)** RDV on RMP bound site and **(C)** RTP on RMP bound site. Protein is represented in new cartoon and white in color. RMP, RDV and RTP are displayed in lime, blue and magenta and rendered in licorice atom-wise.

Molecular docking has revealed the most likely binding pose of RDV and RTP. Targeted docking followed by focused docking has provided their final binding pose at the RMP bound site. The cluster representative of −7.5 kcal/mol and −8.9 kcal/mol docking energy were found for RDV and RTP, respectively (Figure 1A). The superimposition of RMP with docked complex of RDV and RTP has revealed that the obtained poses were close to RMP as the RMSD divergence from the common backbone was less (RDV ∼0.4 Å and RTP ∼1.0 Å) (Figures 1B,C).

### MD Simulation Reveals Stability of Remdesivir monophosphate, Remdesivir, and Remdesivir Triphosphate in SARS-CoV-2 RdRp

**FIGURE 2:**
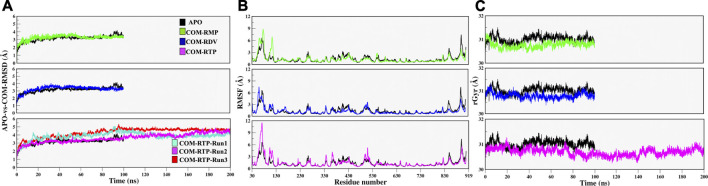
Estimation of systems stability through Molecular Dynamics Simulation over different time scale. **(A)** Root mean square deviation (RMSD) comparison of APO with COM-RMP, COM-RDV and COM-RTP **(B)** Root mean square fluctuations (RMSF) of RdRp residues in different systems, and **(C)** Compactness of all systems assessed through Radius of gyration. All quantitative values are measured in angstrom (Å) unit.

Protein structures must be converged in order to achieve robust results from MD simulations. The quantification of RMSD, RMSF, and Radius of gyrations are good indicators of the overall stability of any protein system. The APO-vs.-COM RMSD comparison reveals that the systems COM-RMP and COM-RDV are achieving stability after ∼20 ns of simulation while system COM-RTP was evolving and did not attain stability initially. The extension of COM-RTP simulation reveals that the system was stable in its next production stretch of 200 ns (Figure 2A). However, to confirm COM-RTP stability, we further decided to perform a triplicate study of this particular system. We found that all the different runs of COM-RTP have shown the same trend where the trajectory was evolving till 80ns while afterwards the system has attained the convergence till 200 ns (Figure 2A). In all systems, the divergence shift from 0 to ∼4 ns is mainly due to the flexible nature of loops (residues 35–100), which has been found to be inconsistent during dynamics. These regions correspond to the NiRAN domain that does not exist in crystals and hence were interpolated during protein preparation. However, their dynamicity is frozen at a certain time-step in all systems, which is satisfactory and eventually makes systems stable (Figure 2A). The RMSF analysis mainly snapped two highly fluctuating regions, Region1-residues 30 to 130, which majorly belong to the interpolated flexible loops of NiRAN domain and another Region2-residues 875 to 900, which is the C-terminal and was also flexible in nature (Figure 2B). In all systems, respective ligands have granted stability to the protein which is reflected in RMSF values, as in many parts COMs are more stable than APO.

**FIGURE 3:**
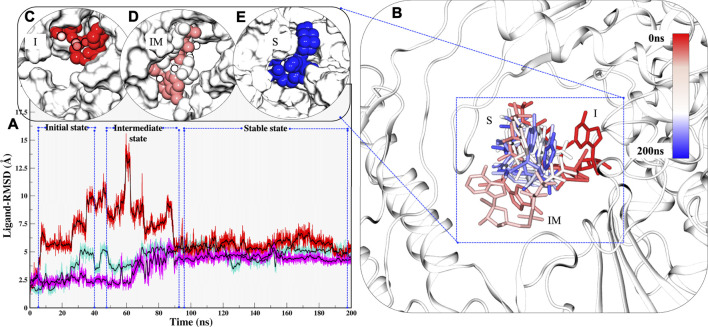
Estimation of different RTP states at MD scale. **(A)** RTP stability in different runs, Run1: Turquoise, Run2: Magenta and Run3: Red. **(B)** Three different states were observed, I: Initial state, IM: Inter-mediate state and S: MD stable state, where RTP is shown in licorice and protein in new cartoon. The conformations of RTP are shown in licorice with time-step coloring method, RTP conformation shown in vdW and protein is represented in surf at **(C)** I state **(D)** IM state and **(E)** S state.

Along with these values, Radius of gyration also highlights that the compactness of COM systems is greater than APO systems (Figure 2C). Further, ligand independent stability was analysed in all systems. The RMP was bound in crystal and its stability was well comprehended by MD simulations (Supplementary Figure S2). We then observed movements of both RDV and RTP. As both were docked poses, their fluctuation was quite justified; however, it was observed that both were trying to gain favorable interactions at respective pockets. We found that RDV achieved complete stability after ∼40 ns (Supplementary Figure S2). A separate introspective RTP stability in triplicate systems was analyzed. In all systems, it was found that RTP’s docked pose was fluctuating initially. However, later it maintained interaction with the Mg^2+^ ion and thus gained stability in the later phase of simulation (Figure 3A). We also curated the movement of RTP throughout the simulation timeline and visualised the same in MD timestep (Figure 3B). We found that RTP movement provided three different states: initial state (I) was the docked pose which, after some time, starts fluctuating and captures itself at intermediate (IM) state and eventually RTP settles itself at its final MD stable state (S) (Figure 3B). We tried to understand the significance of different binding poses at each time step. We found that at I-state RTP is at the catalytic site while at the IM-state RTP is found at the edge of NTP channel (Figures 3C,D). However, MD stable state (S) is found at the centre of NTP channel which might occlude its major portion (Figure 3E) (complete significance of RTP-MD pose is explained in detail in the mechanism of inhibition section). We also found that the ligand RDV found itself at the junction of NTP entry channel and catalytic site while RTP tail favoured NTP entry channel site (explained later). The SASA distribution plot of the binding site reveals that COM-RTP system is least exposed to solvent as compared to other systems (Supplementary Figure S3A). COM-RDV was an exception when compared with APO. This might be attributed to RDV’s self-fluctuation which is higher than RMP and RTP movement in the binding pocket. Adding further insights into ligands’ conduct in the binding pocket, we measured the binding site RMSD. The binding site residues flexibility is reduced upon ligand binding as compared to APO which is a good starting indication as the study cements upon understanding the mechanistic details of the binding pocket (Supplementary Figure S3B). COM-RTP was notably higher as it moved from docked to stable MD pose and gained key interactions (discussed in the next section).

Overall, system stability was asserted through MD simulations, where all systems were stable and special focus was to understand the ligand’s independent behaviour. In continuation, the focus was to highlight the ligand’s integral properties which include key interactions, preferable site, and their respective stability. RDV was fluctuating initially and RMP was most stable. The RTP is given the status of the active form of RDV (Yin et al., 2020) and hence its dynamicity probing is our major concern so that its mechanistic details might be highlighted along with its inhibition mechanism.

### Interaction Fingerprinting of Remdesivir monophosphate, Remdesivir, and Remdesivir Triphosphate Revealed the Key Residues

**FIGURE 4:**
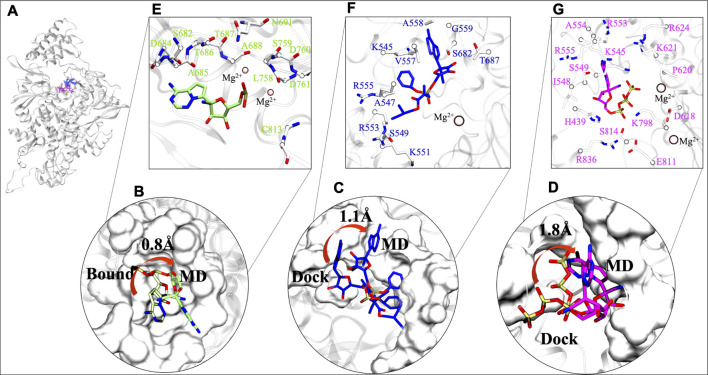
Comparative interaction patterns of RMP, RDV and RTP MD poses. **(A)** MD poses of RMP, RDV and RTP in RdRp. **(B)** RMP’s comparison between its bound and MD representative state **(C,D)** RDV and RTP comparison between docked and MD representative state, respectively. **(E–G)** Arrangement of these molecules at their most stable state, RMP (snapshot: 97.51 ns), RDV (snapshot: 96.31 ns) and RTP (snapshot: 198.56 ns) are lined by respective residues. Interacting residues are shown uniformily in white and licorice representation and small molecules RMP, RDV and RTP in lime, blue and magenta rendered in licorice. Catalytic ion Mg2^+^ is represented in Beads:pink.

The stability of chosen ligands are thoroughly observed by understanding the interaction with residues at their most stable states obtained from their respective MD trajectories (Figure 4A). By comparison from the centre of mass of RMP's bound state to MD state suggests that there is a minor 0.8 Å deviation (Figure 4B). This minor transition was observed due to loss of its interaction with residue K545 (motif F), however, it maintains its interactions with residues of motif B, motif C, and Mg^2+^ ion. It also gains interactions with five other residues, four from motif B and C (D684, A685, T686, L758) and one from motif E (C813) (Supplementary Table S2). Similarly, RDV does a transition of 1.1 Å from docked to MD state (Figure 4C). RDV maintains its interactions with residues of NTP entry channel K545, R553, and R555 and loses its interaction with SDD motif (Figure 4C and Supplementary Table S2). The RDV gains interactions with other motif F residues A547, S549, K551, V557, A558 and G559, as well as with motif B residues S682 and T687. RTP does a shift from its docked pose (Figure 4D) and its RTP-MD state reveals that it majorly forms interactions with all critical residues of motif F and Mg^2+^ ion i.e., K545, R553, and R555. RTP also gains interactions with residues of motif A (D618, P620, K621, and R624) and motif D (K798) which were found missing in its docked state (Supplementary Table S2) and in other systems. The interactions of RTP-MD pose is in accordance with the recently published study by Zhang and Zhou (2020). The importance of these residues is discussed in the section of possible mechanism of inhibition.

After exploring the transitions, we further tried to understand the characteristics of the ligand’s respective binding pocket. So, RMP was chosen as the preferred active site and was surrounded with twelve residues: four hydrophobic residues (A685, A688, L758, and C813), five polar (S682, T686, T687, S691, and S759) and three acidic residues (D684, D760, and D761) (Figure 4E). At its most stable state, RMP is closely surrounded by the SDD motif which forms the base of catalytic activity of RdRp. Further, upon inspection of parent molecules, it has been found that RDV is surrounded by 11 residues and among them, two residues (S682 and T687) have been found in common with RMP (Figure 4F). It is lined with four hydrophobic residues (A547, V557, G559, and V560) as well as two polar (S549 and S682) and has shown interaction with residues K545, K551, R553, and R555. The RDV stable pose reveals that it extends interaction strength at the NTP entry channel. RTP binding pattern reveals that it encompasses residues crucial for NTP entry channel, including three basic residues: K545, R553, and R555 (Figure 4G). Some other residues that surround RTP are H439, I548, S549, A554, D618, P620, K621, R624, K798, E811, S814, and R836 and thus the MD state of RTP appears as a “basic rich pocket” due to the presence of seven basic residues (Figure 4G). The transition of RTP-docked to RTP-MD pose (RMSD 1.8Å) revealed that it prefers NTP channel residues. Hence, the significance of their contribution are assessed further with hydrogen bond (strength and durability) and thermodynamics analysis.

**FIGURE 5:**
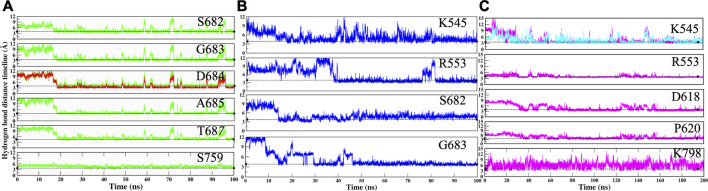
Hydrogen bond analysis throughout MD simulation timeline for **(A)** COM-RMP **(B)** COM-RDV and **(C)** COM-RTP. The dotted lines describe the threshold of 3.5 Å.

The RMP and RTP form six hydrogen bonds, while RDV forms four hydrogen bonds. Residues that were crossing 30% cut-off in hydrogen bond occupancy were considered for this study. RMP forms hydrogen bonds with S682, G683, D684, A685, T687, and S759 (Figure 5A) and their respective occupancies are mentioned in Table 1. Among them, T687 was the lowest contributor with occupancy 33.76% and the rest of the residues formed strong interactions with RMP (Occupancy ∼65–75%) (Table 1). One of the key residues, S759 from the SDD motif, forms a hydrogen bond with RMP, highlighting that RMP and its direct interaction with catalytic sites. RDV forms four hydrogen bonds, where two residues S682 and G683 were in common with RMP, while the other two residues belong to NTP entry channel K545 and R553 (Figure 5B and Table 1). The occupancies of these residues are mentioned in Table 1. The final analysis on RTP reveals that it forms five hydrogen bonds with K545, R553, D618, P620, and K798 (Figure 5C and Table 1) and their respective occupancies are described in Table 1. This shows that the hydrogen bond forming pattern of RMP and RTP is unrelated as RTP has no direct contact with catalytic site residues, while it gains stability by interacting with residues K545 and R553, which are important for NTP entry. This suggests that RTP could be the active form of RDV, possibly binding at NTP entry site instead of other catalytic sites.

**TABLE 1:** Hydrogen bond analysis of COM-RMP, COM-RDV and COM-RTP. The hydrogen bonds with occupancy greater than 30% from the equilibrated trajectory are shown.

Hydrogen bonds of RMP		
Donor	Acceptor	Occupancy (%)
RMP-N5	S682-Main-O	69.20
RMP-N5	G683-Main-N	48.11
RMP-N5	G683-Main-O	72.22
RMP-N5	D684-Main-N	68.36
RMP-N5	D684-Main-O	73.28
RMP-N5	A685-Main-N	71.88
RMP-N5	T687-Side-OG1	33.76
S759-OG	RMP-Side-O4	70.46
**Hydrogen bonds of RDV**		
K545-Side-NZ	RDV-N4	58.20
R553-Side-NH1	RDV-O7	55.52
S682-Side-OG	RDV-O3	59.50
G683-Main-N	RDV-N2	37.14
**Hydrogen bonds of RTP**		
R553-Main-O	RTP-N5	66.8
R553-Main-N	RTP-N5	59.3
K545-Side-NZ	RTP-N3	42.7
RTP-O11	D618-Side-OD1	55.3
RTP-O11	P620-Main-N	57.2
K798-Side-NZ	RTP-O8	59.1

The movements of all ligands can be justified after observing the hydrogen bond distance timeline of each residue (Figure 5). Initially, it was observed that ligands were not forming interactions with crucial residues; however, after they gained interactions, the value shrinks down to ∼3.0 Å. Thus, it can be deduced that by rearranging themselves, the ligands are forming strong interactions at their preferable site, which will pave the way for the identification of key residues and possible mechanisms of inhibition.

Moreover, further thermodynamics analysis forms a strong base and might complement the binding pattern observations obtained from residue mapping and hydrogen bond analysis.

### Binding Free Energy Analysis Reveals Strong Binding of Remdesivir Triphosphate

The speculation over RTP being the active form of RDV has been completely justified by binding free energy analysis. The result indicates that RTP, despite movement, binds stronger than both RMP and RDV. The RTP binds with ∆G_bind_ = −26.37 kcal/mol, which is higher than RDV ∆G_bind_ = −23.83 kcal/mol and RMP ∆G_bind_ = −22.67 kcal/mol (Table 2). This binding pattern was obtained through the PBSA method, while a similar spectrum (RTP > RDV > RMP) was found in the GBSA method as well (Table 2). The difference in the binding energies was due to the higher contribution of the polar solvation energy in the PBSA method. As from Table 2, the electrostatic contribution is more favorable in COM-RMP and COM-RTP than COM-RDV, however was more favorable in COM-RTP.

**TABLE 2:** The calculated binding free energies (kcal/mol) by MM-GBSA/PBSA methods.

Contribution	COM-RMP (kcal/mol)	COM-RDV (kcal/mol)	COM-RTP (kcal/mol)
**∆E**_**int**_	0	0	0
**∆E**_**vdW**_	−41.83	−52.43	−39.11
**∆E**_**ele**_	−102.55	−21.05	−110.57
**∆E**_**GB**_	132.65	53.80	127.86
**∆E**_**surf**_	−4.86	−5.88	−5.44
**∆G**_**gas**_	−144.39	−73.48	−149.68
**∆G**_**solv GB**_	127.78	47.92	122.26
**∆G**_**GB**_	−16.61	−25.55	−27.26
**∆G**_**solv PB**_	121.71	25.85	123.31
**∆E**_**PB**_	124.73	53.83	127.40
**∆E**_**n-polar**_	−3.01	−4.18	−4.08
**∆G**_**PB**_	−22.67	−23.83	−26.36

### Deciphering Residue-Wise Energetic Contributions

The results and observations till now have suggested that findings related to ligand binding have provided a mixed view of the pocket; however, the interaction pattern at stable states explores the key residues. RMP stays at a catalytic site with slight fluctuation, RDV being the bulkiest molecule establishes its interaction from catalytic site to NTP entry channel and RTP, which undergoes rearrangement to establish its prime interaction with residues critically important for NTP entry.

**FIGURE 6:**
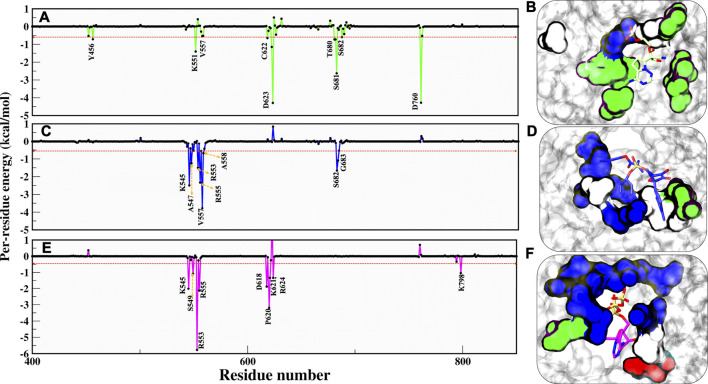
Residue wise decomposition energy (kcal/mol) in all three systems. **(A,C,E)** COM-RMP, COM-RDV and COM-RTP and its respective residue type characteristics is shown panels **(B,D,E)** in surface representation. RMP, RDV and RTP are rendered in licorice and shown in Green, Blue and Magenta respectively. The cut-off energy was -0.5 kcal/mol. Residues chemical properties are shown as: Polar: green, Hydrophobic: white, Basic: blue and Acidic: red.

As per cut-off of −0.5 kcal/mol, RMP contributors D623 and D760 have an upper-bound value of −4.0 kcal/mol, S681 has a mid-bound value of −2.9 kcal/mol, and the remaining lower-bound contributors are Y456, K551, V557, C622, T680, and S682 (Figure 6A). As we have commented earlier on RDV’s fluctuation, we found the same in the energy contribution pattern of residues. Only one residue, V557, has an upper-bound value of −3.8 kcal/mol, while residues K545, R553, R555, and S682 are mid contributors ∼2.0 kcal/mol, and residues A558 and G683 snap at lower-bound (Figure 6B). Eventually, the RTP pattern was analyzed, in which the highest contributor was R553 with −5.9 kcal/mol and residues K545, R555, D618, and P620 had mid-bound value of ∼ −3.0 kcal/mol. At the same time S549, K621, R624, and R798 lie in the lower-bound value (Figure 6C). RTP gets the highest value contribution of ∼ −6.0 kcal/mol, which is missing in both of the ligands. RTP gets its strong binding affinity at the NTP entry channel and strong interactions with K545, R553, and R555 eventually describing its behaviour at the pocket. The comparison between the RTP-docked pose and MD pose shows the gain in stability at the pocket. The major revelation is that at docked pose catalytic site residues D760 and D761 contribute to RTP while at MD pose, those contributions become weak gradually (Supplementary Figure S4). At NTP channel, RTP not only strengthened its interaction with K545 and R553 but also gained interaction with R555 (Supplementary Figure S4). RTP gains four additional interactions (D618, P620, K621, and R624) but also loses five interactions (D452, A547, K551, D760, and D761) and hence we did not find any significant energy change from docked to MD pose (Docked pose ∆G_bind_ = −25.42 kcal/mol and MD pose = −26.37 kcal/mol) (Supplementary Figure S4). It can be clearly seen that the energy contribution of four gained residues is higher than five lost residues (Supplementary Figure S4) and this might be the reason for its stability. Despite the least energy difference, RTP forms strong interaction with NTP channel residues and thus might block the entry of NTP into the active site. As these residues of motif F interact with primer strand RNA and stabilize the incoming nucleotide (Yin et al., 2020), RTP might be destabilizing this interaction.

Additionally, we have characterized the pocket based on contributing residues and its respective chemical nature that might be crucial for designing ligands. Falling from RMP to RTP, it can be observed that RTP site (MD pose) is “basic rich” as residues K545, R553, R555, K621, R624, K798, and R836 are the significant contributors (Figures 6D–F) while in the other two systems this uniformity is not found. This makes the RTP site unique and relevant for designing more potential inhibitors.

### Charge Complementarity Profile

**FIGURE 7:**
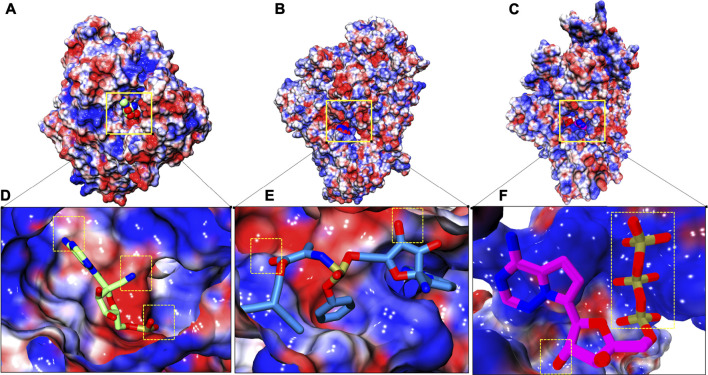
Electrostatic surface complementarity. **(A–C)** Electrostatic potential isosurface of whole complexes: COM-RMP, COM-RDV and COM-RTP respectively and site of embedded small molecules is highlighted in solid yellow box **(D–F)** Respective binding site snapshot of systems in aforementioned order. Positive and negative electrostatic potential is rendered in blue and red, respectively. Dotted yellow boxes show positive complementarity patches observed at their respective stable states.

Electrostatic potential was evaluated for all three systems in order to understand electrostatic complementarity between ligands and protein (Figures 7A–C). Complementarity profile is expressed in charged units, indicating a spectrum of optimal complementary charges preferred by protein along its molecular surface and afterwards its complementarity is established with respect to ligand. RTP gains three slightly favorable patches with its two ‘N’ atoms and two ‘O’ atoms (Figure 7D), while RDV gains only two small complement patches (Figure 7E). Unlike these two, RTP phosphate tail finds a large complementary patch with its respective pocket along with a slightly favorable patch with its ‘O’ atom (Figure 7F).

The final protein-ligand analysis justifies the RTP stability at NTP entry channel as it gains perfect complementarity from protein while the other two clearly miss this gain from their respective preferable site. This analysis also shows that RTP-MD pose is the best pose.

### The Free Energy Landscape Undermines the Thermodynamically Most Stable Basin of Remdesivir Triphosphate

**FIGURE 8:**
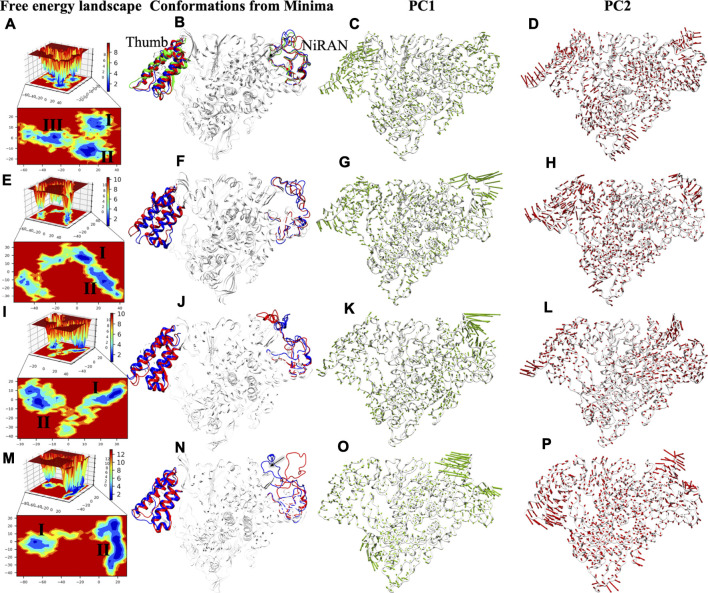
FEL analysis and porcupine plots of SARS-CoV-2 RdRp in bound and unbound states. The panels **(A,E,I,M)** of 2D and 3D free energy landscape (FEL) plots between PC1 (*x*-axis) and PC2 (*y*-axis) for APO, COM-RMP, COM-RDV and COM-RTP respectively. The color bar represents the Gibbs free energies in the plot ranging the lowest energy (blue) to highest energy (red) conformation states. The low energy minima states are shown in the 2D FEL plots. The minima extracted from FEL plots are superimposed for all simulated structures depicting the conformational changes in the panels **(B,F,J,N)**. The red, blue and green conformations represent the minima I-III respectively in the panels **(B,F,J,N)**. Porcupine plots generated using extreme PC1 panels **(C,G,K,O)** and PC2 projections panels **(D,H,L,P)** for all the four simulated systems. The direction of the green and red arrows (in PC1 and PC2 respectively) at each Cα shows its direction of motion and the length of arrow depicts its strength. The protein is represented in tube form and colored in white. The regions that are highly fluctuating are labelled.

To capture the significant conformational changes, PCA was applied to all the simulated systems. The first two PCs were found as important components for capturing the maximum variance in the Cα displacement during the MD simulations. This was found on inspection of the path of the trajectory connecting the minima’s and the sub-conformational spaces of different systems. The minima are numbered as per the appearance of the basins w.r.t. time during MD simulations. We found three minimas in APO systems, while only two minimas are observed in all complex systems (Figure 8). APO systems show large areas of conformational space that have three minima at ∼50 ns, ∼60 ns, and ∼80 ns and among them, the deepest minima was observed at 80 ns (Figure 8A). Upon superimposition of the conformations extracted from these basins, the structural changes were observed, which may be important for RdRp activity. The interpolated loop (residue 51–83) at NiRAN domain and helix of thumb (residue 832–895) have displayed the major fluctuations while other regions are well-aligned (Figure 8B). The loops of NiRAN domain have shown bidirectional movement, which has outward (at ∼50 ns) to the inward movement (at ∼80 ns) as trajectory tends towards stability (Figure 8B), while thumb helices show slight dislocation at every minima step (Figure 8B). The porcupine plot of PC1 and PC2 shows highest atomic fluctuations in the above-mentioned regions as well (Figures 8C,D). The variation captured by PC1 and PC2 is correlated with the RMSF values of these regions (Figure 2B). COM-RMP has two minima at ∼75 and ∼95 ns respectively (Figure 8E). The structural changes were the same as in APO (Figure 8F) but in this case, the loop of NiRAN domain does not move inwards and in both the basins it has shown outward movement (Figure 8F). PC1 and PC2 captured fluctuations in the same regions in COM-RMP. The atomistic fluctuation of NiRAN domain at PC1 shows higher fluctuation than APO, while the rest have shown this with lesser magnitude (Figures 8G,H). COM-RDV also displays two minima: one minima at ∼50 ns and the other at ∼95 ns (Figure 8I). The second minima were broader and deeper as compared to minima I (Figure 8J). Similar observations were found as of COM-RMP, which depicts the outward movement of NiRAN domain loop along with the changes in helices of thumb subdomain (Figure 8J). PC1 and PC2 capture the highest atomistic fluctuation at similar regions (Figures 8K,L). The most different trends were observed in the case of COM-RTP. Upon landscape inspection, we found that COM-RTP surpassed all systems with broader and deepest minima at ∼160 ns (Figure 8M). As such deep minima were missing in other systems, COM-RTP achieves its lowest free energy state. Another difference is the inward movement of NiRAN domain loop from outward movement (Figure 8N). This is one of the states that was snapped in APO trajectory (previously mentioned). The equilibrium shift from minima I (at ∼50 ns) to minima II (at ∼160 ns) has elucidated the lowest free energy state of RdRp in RTP bound condition (Figure 8N). Another feature that has been observed is the minimization of thumb domain fluctuation while in previous systems these fluctuations were notable (Figure 8O). Also, the directionality of the projections at PC1 of NiRAN domain changes to inward, while in other systems it was outward (Figure 8O). Similarly, projections of the helices of thumb domain were also inward as shown by PC2 (Figure 8P). The inward movement COM-RTP shows that RTP binding at palm sub-domain might have an impact on internal wiring as it changes the atomistic fluctuation of distal NiRAN domain. Since NiRAN and palm domain share close proximity and may engage in a functional interaction (Zhang et al., 2020), the strong binding of RTP at NTP entry channel might be justified.

Overall, COM-RTP displays different behavior as compared to RMP/RDV systems. Two major regions have been identified through PCA and FEL analysis. Thumb subdomain is crucial for template entry while NiRAN domain is known for its interaction with other protein partners (Zhang et al., 2020). COM-RTP follows one trend of APO, where the inward movement of the loop was snapped while all other systems follow the either trend of APO i.e. outward movement. One of the major findings observed from PCA and FEL analysis is the deepest basin obtained in system COM-RTP which justifies the impact of RTP binding on protein significantly. The presence of RTP has not only minimized the nearby regions (thumb domain helices) but also led to significant changes in the NiRAN domain which may have caused an allosteric regulatory effect. Thumb domain acts as a door for NTP entry, and our observation does suggest that it was completely minimized in COM-RTP, thus these changes may justify the role of RTP rearrangement at NTP entry site. In continuation, it may not allow the entry of new nucleotides, which might lead to inhibition of replication.

### Computational Alanine Scanning

The key residues K545, R553, and R555 that are crucial for NTP entry channel have shown a remarkable drop in their contribution to RTP stability after they were mutated to alanine (Supplementary Figure S5). Residue P620 that has shown backbone displacement to rigidify the channel was also considered and it was found that it has a slight contribution. Residues of catalytic site S549, D760, and D761 were also considered to justify the RTP binding at NTP channel, and found that their contribution is negligible. These observations further confirm the criticality of NTP facilitators residues (K545, R553 and R555) and also justify the significance of RTP MD pose.

### Mapping of Potential Residues of SARS-CoV-2 RdRp Protein With the Allosteric Sites of Other Viruses

Since RdRps are structurally and functionally well conserved, we have mapped the possible allosteric sites for SARS-CoV-2 RdRp based on literature and crystallographic data of RdRp of HCV (Mittal et al., 2020), BVDV (Mittal et al., 2019), and DENV (Maddipati et al., 2020). Allosteric inhibitors customize the internal motions and conformational states of the enzyme and are very specific to a target, thus avoiding off-target effects (Davis et al., 2016; Mittal et al., 2020). Although the sequence and structural similarity among their sequences with CoV-2 RdRp is less than 30%, the secondary structures and functional motifs are well conserved. Therefore, we found four probable allosteric sites based on structural alignment with HCV RdRp: T1, P1, P2, and TES sites on CoV-2 RdRp.

**FIGURE 9:**
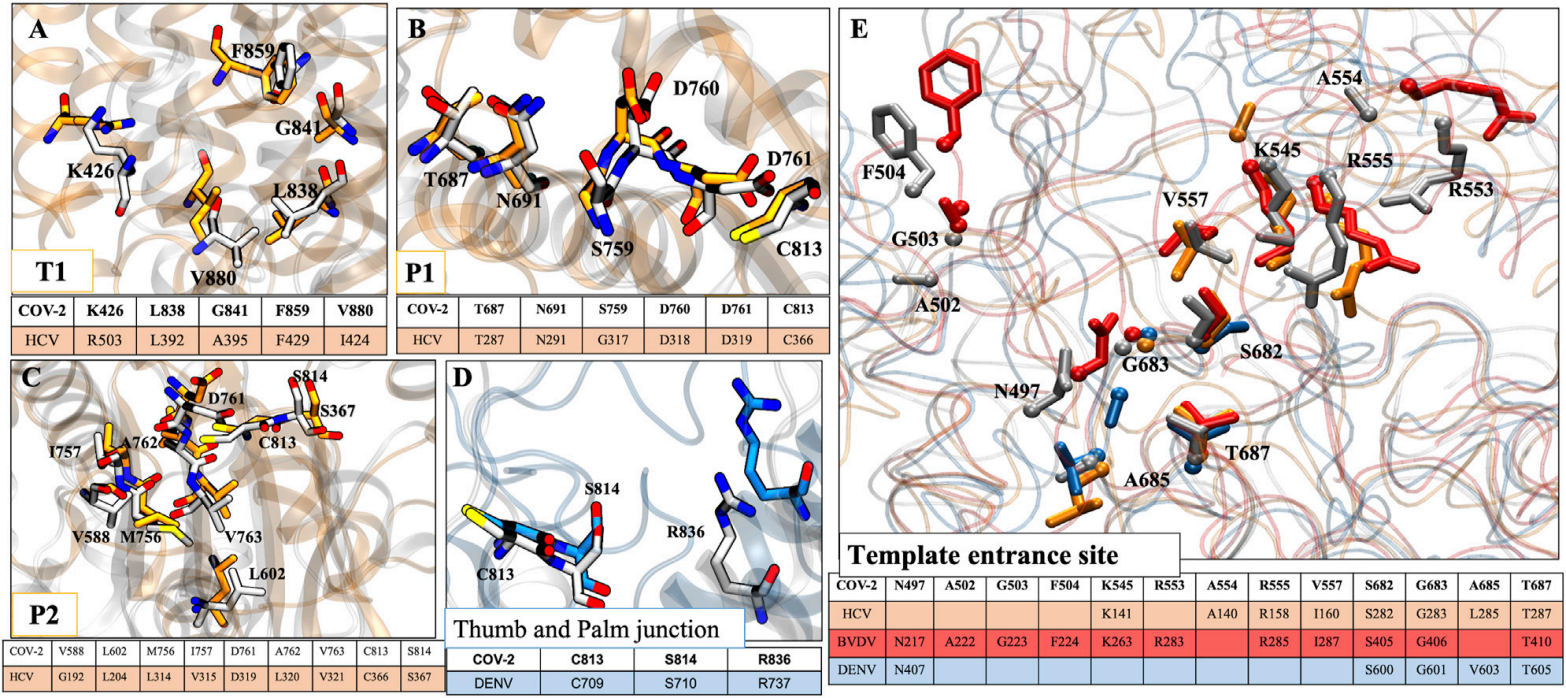
Conservity analysis of allosteric sites in different viral RdRp’s. The panels **(A–C)** represent three conserved allosteric sites in HCV and CoV2 RdRp. Panel **(D)** represents conserved allosteric sites in DENV and CoV2 RdRp. **(E)** The conservation analysis of template entrance site (TES) in CoV-2, HCV, BVDV and DENV. For figure clarity, only sidechain and Cα atoms of residues are shown in this panel. The respective tables at every panels corresponds to conserved residues obtained after structural alignment. Residues color coding is done as follows: HCV: orange, CoV2: white, DENV: blue, BVDV: red.

*T1 site*: The general APO HCV RdRp consists of a Δ1 loop tucked on the thumb domain which is displaced by non-nucleoside inhibitors (NNIs) and forms a hydrophobic T1 pocket (Mittal et al., 2020). In CoV-2 RdRp also, we observed the same Δ1 loop protruding from the finger domain and extends till thumb domain. Along with this, the residues K426, L838, G841, F849, and V880 of CoV-2 RdRp are structurally aligned with the residues R503, L392, A395, F429, and I424 of HCV RdRp but the proper pocket surface is not present (Figure 9A). So, we believe that if NNIs are designed for this site based on the aforementioned residues this may result in a similar mechanism of inhibition by causing displacement of the Δ1 loop, which will disrupt the functionality of the RdRp.
*T2 site*: As per the T2 site of HCV RdRp (Mittal et al., 2020) and allosteric site 1 of DENV RdRp (Maddipati et al., 2020) below the thumb domain and C-terminal, we did not find any relevant or conserved residues in CoV-2 RdRp.
*P1 site:* As per the P1 site of HCV RdRp (present adjacent to the active site on palm subdomain) (Mittal et al., 2020), we found residues T687, N691, S759, D760, D761, and C813 of CoV-2 RdRp as structurally well-conserved residues with their corresponding residues T287, N291, G317, D318, D319, and C366 in HCV RdRp (Figure 9B). Therefore, the NNIs designed at this site could interfere with the active site mechanism as well as incoming NTPs.
*P2 site*: The residues V588, L602, M756, I757, D761, A762, V763, C813, and S814 of CoV-2 RdRp are found to be aligned with the residues G192, L204, L314, V315, D319, L320, V321, C366, and S367 of HCV RdRp (Figure 9C). Although a similar groove of P2 pocket of HCV is not observed in CoV-2 RdRp, the possibility of pocket formation by NNIs is still there that may interfere with the NTP entry into the RdRp.

As per DENV RdRp’s allosteric site 2 (interface/junction between thumb and palm or N-pocket) (Hilgenfeld and Vasudevan, 2018; Maddipati et al., 2020), we found three conserved residues, C813, S814, and R836 in CoV-2 RdRp also (Figure 9D). Therefore, the NNIs designed for this site would possibly interrupt the *de novo* initiation activity of the viral RdRp (Hilgenfeld and Vasudevan, 2018).

*TES site:* Since the RNA template entrance site (TES) exists in all polymerases and has been well explored in DENV, BVDV, and HCV RdRps, this site could also be targeted for designing NNIs. We have aligned all these four RdRps and highlighted the conserved residues accordingly. We found more number of conserved residues in BVDV RdRp than others with CoV-2 RdRp. In CoV-2, the conserved residues are N497, A502, G503, F504, K545, R553, A554, R555, V557, S682, G683, A685, and T687 (Figure 9E). Among these residues, K545, R553, and R555 are involved in escorting the RNA template and positioning of NTPs at the catalytic site.

Further, we implemented an unbiased and ligand-independent approach to search for potential druggable binding sites in CoV-2 RdRp by using the SiteMap module of Schrodinger. We identified 4 out of 5 sites that could be targeted for designing small molecules. As per the highest Dscore, the best site is site_3 which corroborates with DENV RdRp N-pocket and HCV P1 site. The site_1 and site_4 correspond to the template entrance channel while site_4 corresponds to the P2 site as HCV RdRp (Supplementary Figure S6). The residues lining these sites are mentioned in Supplementary Table S3. Therefore, the overall analysis hints for these allosteric sites for rational or targeted designing of NNIs for SARS-CoV-2 RdRp.

The conservity analysis revealed the significance of all sites to develop NNIs, however the importance of TES as allosteric site and its lining residue is well documented. The significance of these residues, especially K545, R553, and R555, were well aligned with our simulation studies as well, which justifies the RTP binding and its probable inhibition mechanism. Thus, a comprehensive biochemical and structural-dynamics studies of this enzyme may reveal more insights about other potential druggable allosteric sites (Shi et al., 2020).

### Possible Mechanism of Inhibition by Remdesivir Triphosphate

**FIGURE 10:**
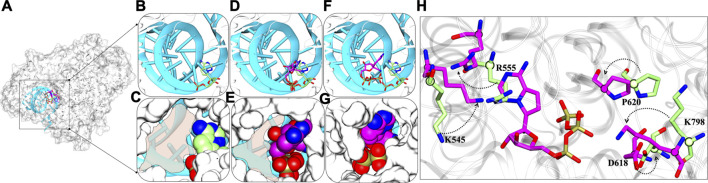
Dynamic insights at NTP entry site **(A)** Superimposed structure of RMP-bound with RTP-docked and RTP-MD pose in the presence of primer-template strand. The inset shows the RMP bound, RTP docked and RTP MD poses in the panels **(B,D,F)** in licorice representation and primer-template strand in cyan New Cartoon representation. The panels **(C,E,G)** show the NTP channel occlusion by these poses with protein rendered in white surface representation and molecules in vdW representation. **(H)** Crucial residues with major backbone displacement are highlighted that rigidifies the channel in the presence of RTP-MD pose. RMP lined residues bound with RMP are shown in Licorice: lime and RTP lined residues are in Licorice: magenta. The transparent shading represents the available space at NTP entry site. The direction of arrows represents the transition of residues from crystal RMP-bound pose to RTP-MD stable pose.

The detailed possible mode of inhibitions in RdRps were explored and based on various analysis, the role of RTP at NTP entry site looks promising for the inhibition of RdRp and thus we proposed the possible inhibition mechanism. We compared the RMP-bound pose with RTP- RTP-MD pose that provided significant information and thus support RTP rearrangement at NTP entrance site (Figure 10A). At RMP-bound pose, it can be clearly seen that in this binding mode there is available space for NTP to enter into the cavity (Figures 10B,C) whereas a similar trend was found with RTP-docked pose as well (Figures 10D,E). The most distinguished feature was observed in RTP-MD pose, extracted from the deepest basin shows that RTP occupies the space completely and therefore occludes the NTP entry channel (Figures 10F,G). We obtained a new set of residues when the transition of RTP occurs from dock to MD binding pose (Figure 4D and Supplementary Table S2). Out of those set of residues, D618, P620, and K798 contributed to RTP stability as observed in thermodynamics analysis (Figure 6). Hence, we tried to elucidate their importance and upon inspection, we found that residues K545, D618, P620, and K798 showed backbone displacement towards the NTP entry channel to rigidify the channel and provide tight packing to RTP. Along with these R555 also undergoes backbone displacement which helps to accommodate RTP at this channel. Furthermore, the residues H439, R836, and S814 do not contribute much energetically, however, their presence at the periphery of the channel does provide the additional rigidity to the RTP pocket.

Thus, RTP’s pose explored through MD simulations embraces the NTP entry channel dynamicity, by which it occupies a complete area that is left open in case of RMP and RTP-docked pose. It can also be estimated that NTP would be now sterically hindered due to non-availability of space at the channel and the change in conformations of the key residues essential to escort NTPs into the polymerase would be possible to perturb the replication process.

## Conclusion

In this work, a thorough comparative study between RDV (parent molecule) and its metabolites, RMP (crystal bound), and RTP (active form) was performed to understand the mechanism of RdRp inhibition, the reason behind the active form of RTP only, and its interaction pattern with key residues. Previous studies with RTP or RDV were performed on homology models and after the arrival of crystal structure of SARS-CoV-2 RdRp, we implemented a holistic approach to extract the meaningful insights that lead to inhibition. RMP was bound in crystal, however as stated by Yin et al. in 2020, the metabolite RTP is known to be the active form of RDV (Yin et al., 2020). Therefore, to explore the dynamic spectrum of the binding pocket, we included both the metabolites and parent molecules to compare the binding pattern. Molecular docking of RDV and RTP provided the initial information of RTP with highest docking energy being the best candidate in this competitive analysis. The MD simulations revealed that all systems are stable throughout the trajectory, however, the RTP has shown movement at initial states in the binding pocket, but after gaining the interaction with residues and Mg^2+^ ion, it achieved the most stable state. We observed that the most stable state obtained from MD is not the initial docked-pose. Thermodynamics analysis revealed that RTP specifically interacts with the residues K545, R553, and R555, important for NTP entry (Yin et al., 2020; Zhang and Zhou, 2020). This outcome correlates well with our finding. In RTP interaction map, four other basic residues, K621, R624, K798, and R836, were found trapped in the “basic rich pocket”. Apart from these we identified four new residues, S549, D618, P620, and K798, as the key contributors to RTP binding in the terms of energetic analysis. The conformational analysis reveal that their backbone movement facilitates the tight packing of RTP at NTP channel. Overall the combination of motif A, E, and F residues are involved in the interaction with RTP supports the possible inhibition mechanism. PCA analysis elucidates two key regions, i.e. thumb and interpolated loops of NiRAN domains, that have shown high atomistic fluctuations. Atomistic fluctuations were merely observed in thumb regions which might be because of the rearrangement of RTP. As thumb domain is adjacent to template entry site and its structural minimization upon RTP binding lays down the possibility that it possibly hinders the template entry and perturbs the replication process. To speculate the possible inhibition, the conservity analysis of different allosteric sites of RdRps of HCV, BVDV, DENV, and SARS-CoV-2 highlighted the potential allosteric sites apart from RTP’s NTP site. Overall, the comparative analysis and computational insights encourage to target these residues at NTP entrance site for the discovery of non-nucleosidic antiviral inhibitors.

## Data Availability

The original contributions presented in the study are included in the article/Supplementary Material, further inquiries can be directed to the corresponding author.
