# Supplementary material for: Molecular Dynamics Simulations Reveal the Interaction Fingerprint of Remdesivir Triphosphate Pivotal in Allosteric Regulation of SARS-CoV-2 RdRp
Source: Front Mol Biosci. 2021 Aug 20;8:639614. doi: 10.3389/fmolb.2021.639614 (PMC8417884; doi:10.3389/fmolb.2021.639614)
Supplement: Supplementary file 1 [file DataSheet1.docx]

Supplementary Material for “**Molecular dynamics simulations reveal the interaction fingerprints of Remdesivir triphosphate crucial in allosteric regulation of SARS-CoV-2 RdRp”**

**Mitul Srivastava^#^, Lovika Mittal^#^, Anita Kumari^#,^ Shailendra Asthana***

Translational Health Science and technology Institute (THSTI), Haryana, 121001, India

#These authors contributed equally

*To whom the correspondence should be addressed.

Dr. Shailendra Asthana

Principal Scientist

Translational Health Science and Technology Institute,

NCR Biotech Science Cluster, 3rd Milestone, Faridabad-Gurugram Expressway, Faridabad, Haryana, India-121001

Email: sasthana@thsti.res.in

**Supplementary Figures**

**
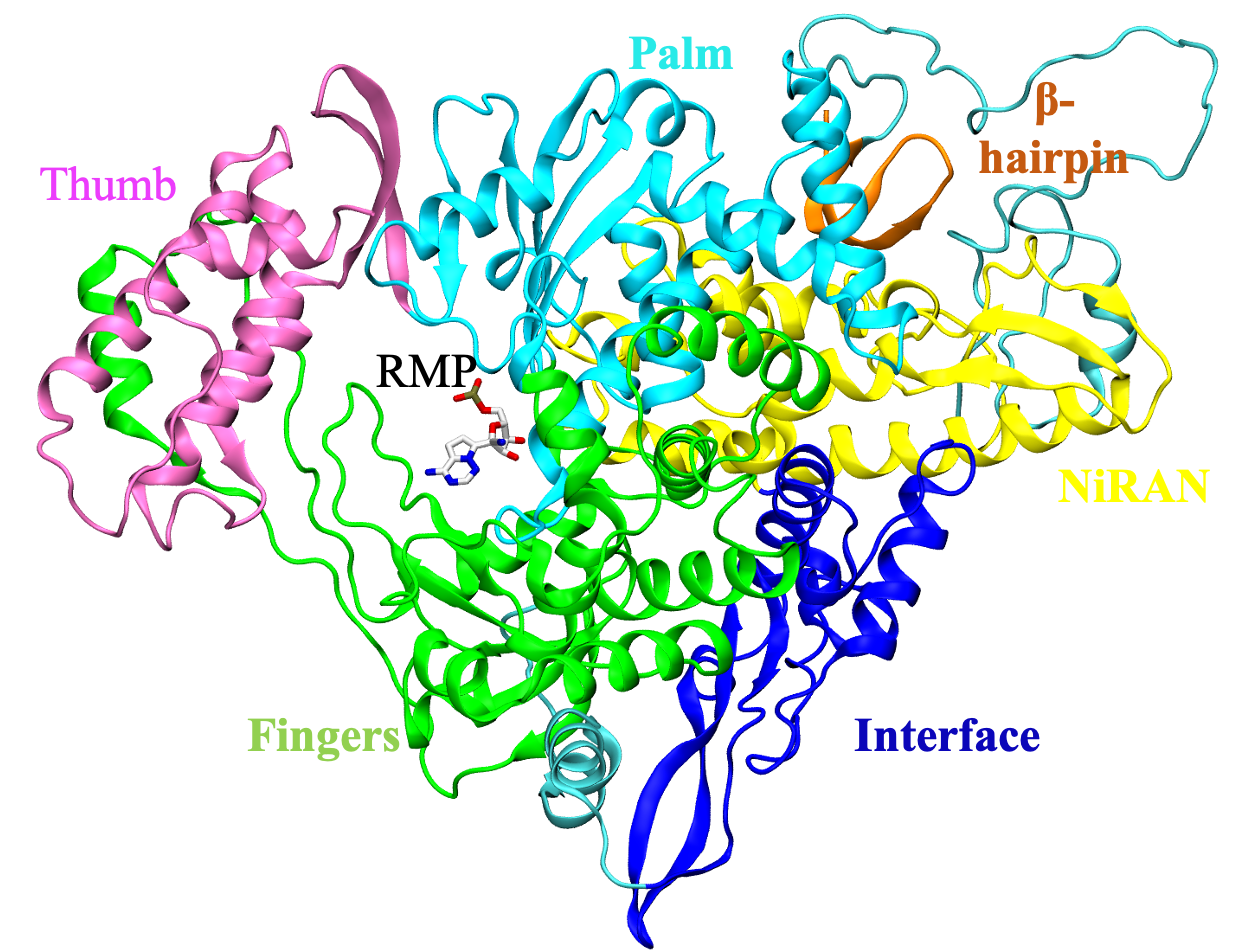
**

**Supplementary Figure S1.** Overall architecture of SARS-COV2-RdRp

**
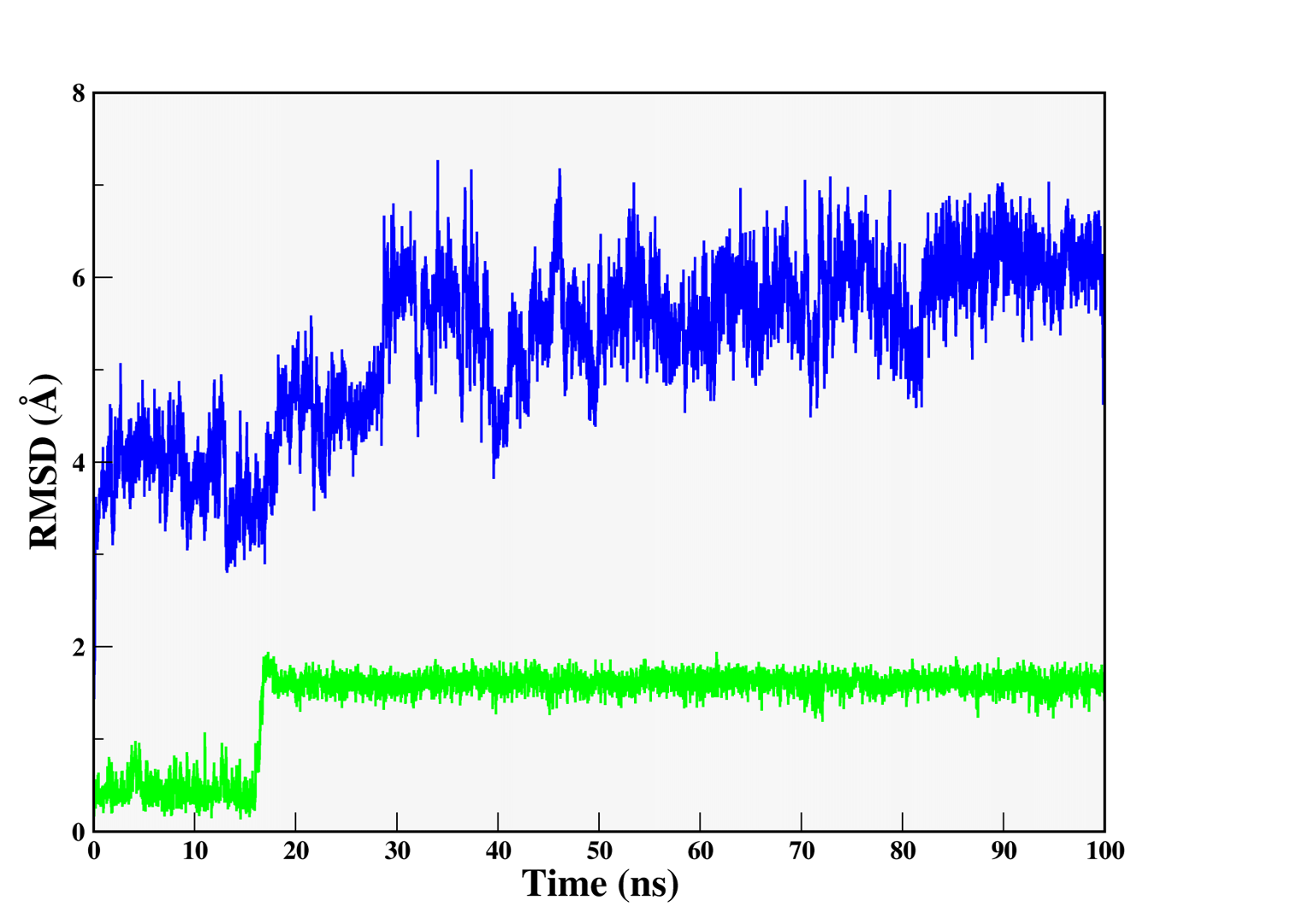
**

**Supplementary Figure S2.** RMP and RDV ligand stability over MD time scale. Green: RMP and Blue: RDV


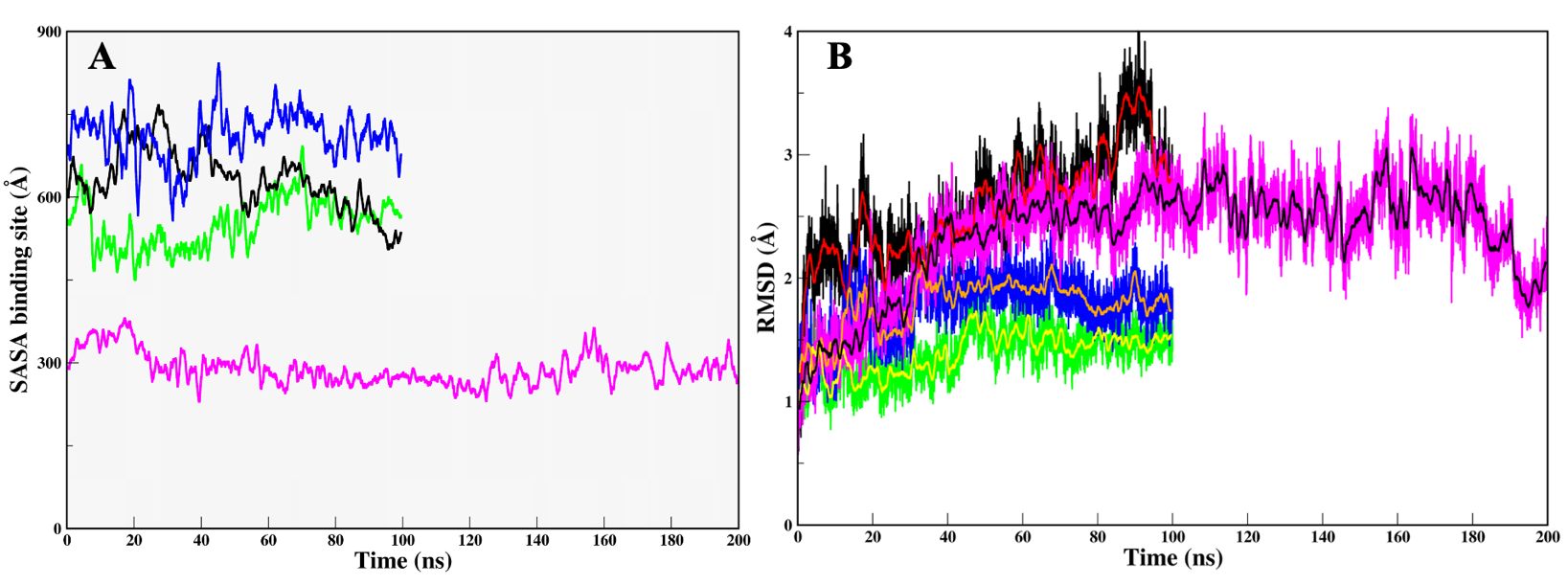


**Supplementary Figure S3.** (A) SASA binding site over MD simulation of APO, COM-RMP, COM-RDV and COM-RTP and (B) Root mean square deviation (RMSD) of binding site of above-mentioned systems. APO: Black, Green: COM-RMP, Blue: COM-RDV and Magenta: COM-RTP


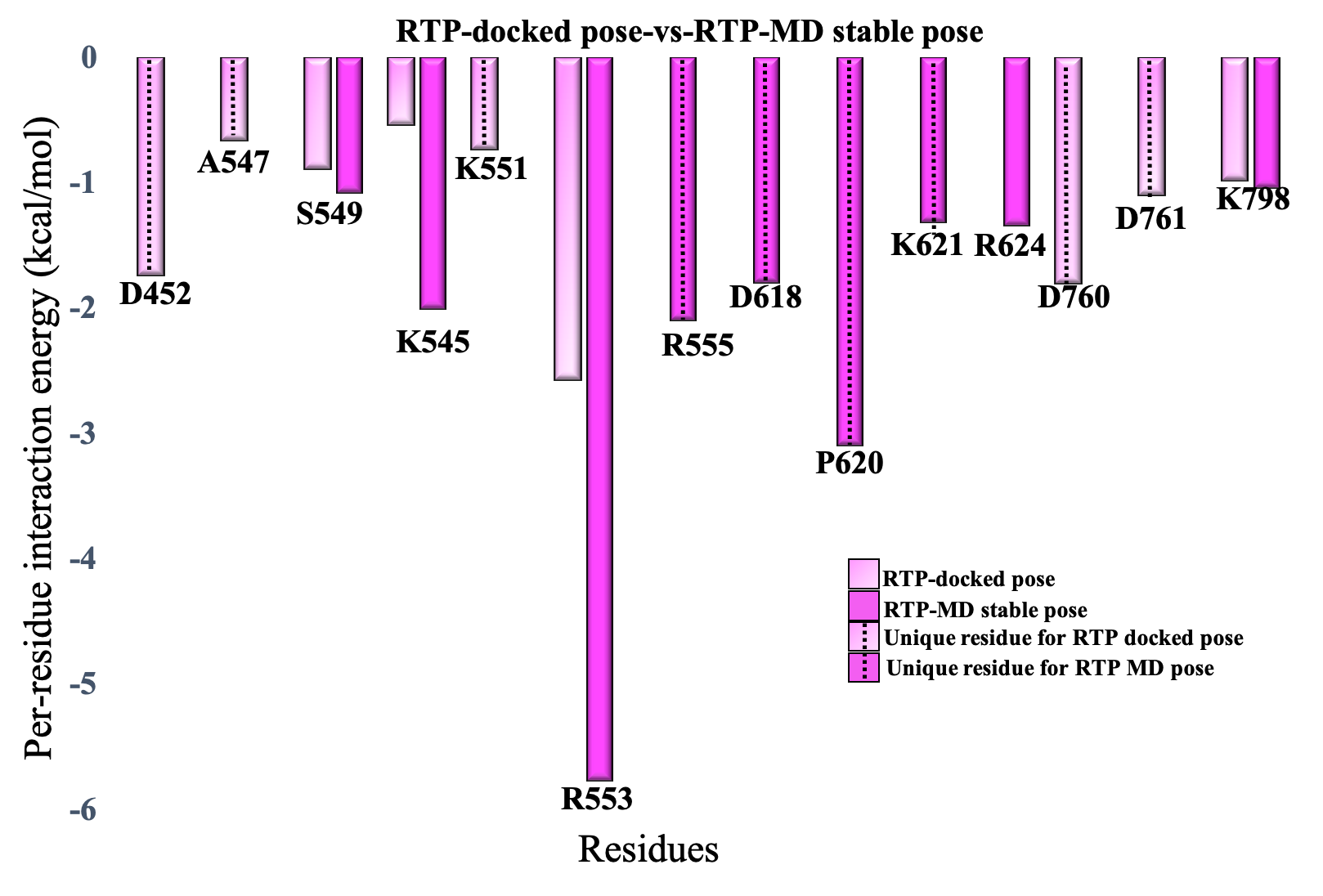


**Supplementary Figure S4.** Comparison of RTP-docked pose and RTP-MD stable pose on the basis of energetic contribution


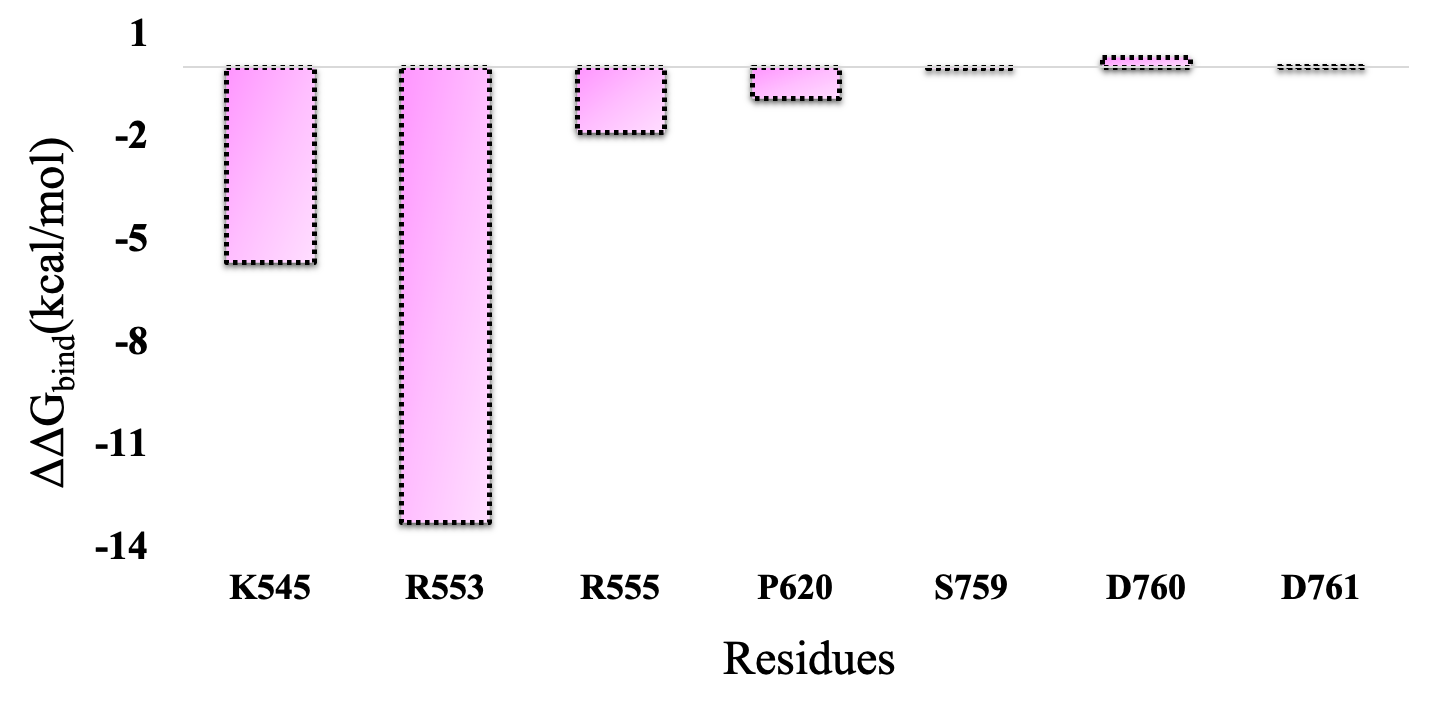


**Supplementary Figure S5**. Computational alanine scanning


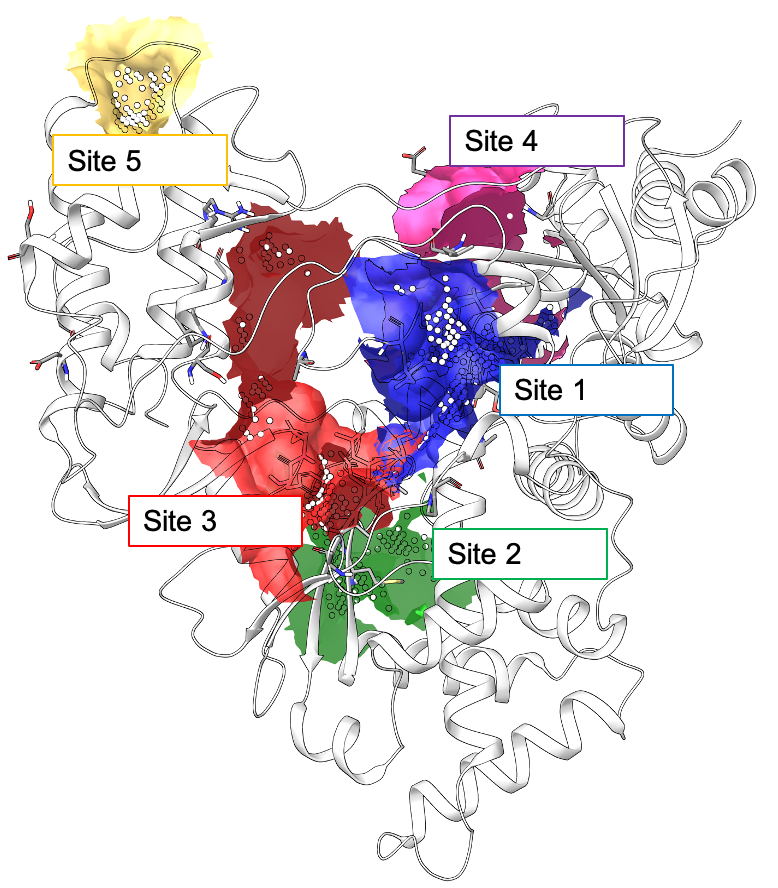


**Supplementary Figure S6.** Ligand independent site search using SiteMap. Site1 to Site5 are marked in blue, green, red, magenta and yellow respectively.

**Supplementary Tables**

**Supplementary Table S1.** Simulation details of each system

| **ID** | **Systems** | **Time**  **(ns)** | **Total no. of atoms** | **Water**  **molecules** | **Ions** |
| --- | --- | --- | --- | --- | --- |
| 1 | APO | 100 | 137617 | 41156 | 10 Na^+^ |
| 2 | COM-RMP | 100 | 135694 | 40341 | 6 Na^+^ |
| 3 | COM-RDV | 100 | 135698 | 40501 | 5 Na^+^ |
| 4 | COM-RTP | 200 x 3 | 135196 | 40278 | 6 Na^+^ |

**Supplementary Table S2.** Interaction fingerprinting from Bound/Docked to MD stable state in COM- RMP, COM-RDV and COM-RTP systems.

| **Static state -vs.- MD state (<=5.0Å)** | | | | | | |
| --- | --- | --- | --- | --- | --- | --- |
| **Motifs/regions** | **RMP** | | **RDV** | | **RTP** | |
|  | **Bound** | **MD** | **Dock** | **MD** | **Dock** | **MD** |
|  |  |  |  |  |  | *H439* |
|  |  |  | D452 | *-* |  |  |
| **motif F** | K545 | *-* | K545 | *-* | K545 | *K545* |
|  |  |  |  |  | I548 | *I548* |
|  |  |  |  | *S549* | S549 | *S549* |
|  |  |  |  | *K551* | K551 | *-* |
|  |  |  |  |  |  |  |
|  |  |  | R553 | *R553* | R553 | *R553* |
|  |  |  |  |  |  | *A554* |
|  | R555 | *-* | R555 | *R555* | - | *R555* |
|  |  |  | T556 |  | - |  |
|  | V557 | *-* | V557 | *V557* |  |  |
|  |  |  |  | *A548* |  |  |
|  |  |  |  | *G559* |  |  |
| **motif A** |  |  |  |  | - | *D618* |
|  |  |  |  |  | - | *P620* |
|  |  |  |  |  | - | *K621* |
|  | C622 | *-* |  |  |  |  |
|  | D623 | *-* | D623 | *-* |  |  |
|  |  |  | R624 |  | - | *R624* |
| **motif B** | T680 | *-* |  |  |  |  |
|  | S682 | *S682* | S682 | *S682* |  |  |
|  | - | *D684* |  |  |  |  |
|  | - | *A685* |  |  |  |  |
|  | - | *T686* |  |  |  |  |
|  | T687 | *T687* | T687 | *T687* |  |  |
|  | A688 | *A688* | A688 |  |  |  |
|  | N691 | *N691* | N691 |  |  |  |
| **motif C** | - | *L758* |  |  | L758 | *-* |
|  | S759 | *S759* | S759 | *-* | S759 | *-* |
|  | D760 | *D760* | D760 | *-* | D760 |  |
|  | D761 | *D761* | D761 | *-* | D761 | *-* |
|  |  |  |  |  | - | *K798* |
| **motif E** |  |  |  |  |  | *E811* |
|  | - | *C813* | - | *C813* | C813 | *-* |
|  |  |  |  |  | S814 | *S814* |
|  |  |  |  |  |  | *R836* |

**Supplementary Table S3.** The conserved residues elucidated after structural alignment are shown.

| **Title** | **SiteScore** | **size** | **Dscore** | **volume** | **exposure** | **enclosure** | **contact** | **phobic** | **philic** | **balance** | **don/acc** | **residues** |
| --- | --- | --- | --- | --- | --- | --- | --- | --- | --- | --- | --- | --- |
| site_4 | 1.015 | 97 | 0.854 | 320.362 | 0.578 | 0.738 | 0.964 | 0.056 | 1.579 | 0.035 | 0.383 | Chain A: I495-G504, A513, Y517, Q542-N544, V558-I563, T566, N569, R570, Q574, L577, K578, G684-A686 |
| site_1 | 1.015 | 134 | 0.877 | 283.661 | 0.54 | 0.72 | 0.973 | 0.137 | 1.518 | 0.09 | 0.553 | Chain A: A444-D446, A449, S452, D453, Y456, Y457, T541, M543, K546, N553-A559, Y620-R625, E666, V668, M677, T681-S683, T688, N692 |
| site_2 | 0.994 | 93 | 0.892 | 191.737 | 0.505 | 0.727 | 1.05 | 0.119 | 1.382 | 0.086 | 1.47 | Chain A: K479-Y484, R584-V589, N601, M602, T605, V606, S608, Y747, L750, R751, F754-M757 |
| site_3 | 0.963 | 106 | 0.919 | 296.009 | 0.726 | 0.642 | 0.777 | 0.139 | 1.248 | 0.112 | 0.823 | Chain A: F442, A548, I549, G617-Y620, D761-A763, A798-W801, H811-Q816, P833, D834, R837, I838, A841, F844-D846, I848, R859, L863 |
| site_5 | 0.82 | 50 | 0.853 | 118.335 | 0.664 | 0.604 | 0.707 | 0.756 | 0.535 | 1.413 | 2.06 | Chain A: D852-T854, M856, I857, L892, E895,896,897,898,899,900 |
